# Machine Learning in Radiation Oncology: Opportunities, Requirements, and Needs

**DOI:** 10.3389/fonc.2018.00110

**Published:** 2018-04-17

**Authors:** Mary Feng, Gilmer Valdes, Nayha Dixit, Timothy D. Solberg

**Affiliations:** Department of Radiation Oncology, University of California San Francisco, San Francisco, CA, United States

**Keywords:** machine learning, radiation oncology, big data, predictive models, process improvement

## Abstract

Machine learning (ML) has the potential to revolutionize the field of radiation oncology, but there is much work to be done. In this article, we approach the radiotherapy process from a workflow perspective, identifying specific areas where a data-centric approach using ML could improve the quality and efficiency of patient care. We highlight areas where ML has already been used, and identify areas where we should invest additional resources. We believe that this article can serve as a guide for both clinicians and researchers to start discussing issues that must be addressed in a timely manner.

## Introduction

The expanding collection and sharing of data, increases in computational power, and perhaps most significantly, advances in machine learning (ML) and artificial intelligence, are rapidly transforming society, and offer the potential for similar transformation within health care. Ongoing advances in ML and big data analytics have spurred numerous efforts in precision oncology (1, 2), and the field of radiation oncology is uniquely poised to benefit from prudent application of such techniques. Radiation oncology has many specific challenges, however, ranging from unique datasets [e.g., 4DCT, CBCT, dose, structures, setup, and quality assurance (QA) information], limited clinical outcomes data, variation in dose and fraction schedules comprising standard of care, interaction of radiation and chemotherapy, limited access to genomics data, and other complexities. The historical reliance on empirical approaches such as the linear-quadratic model further influences clinical practice (3). Furthermore, the current ML hype is largely a result of success in a few, very specific tasks, such as image classifications, games, and autonomous driving systems (4–6). It is critical that we understand this success depends as much on the nature of the task as on the nature of the algorithm and the availability and quality of data, and thus meaningful gains in our field may prove more challenging.

**Figure 1:**
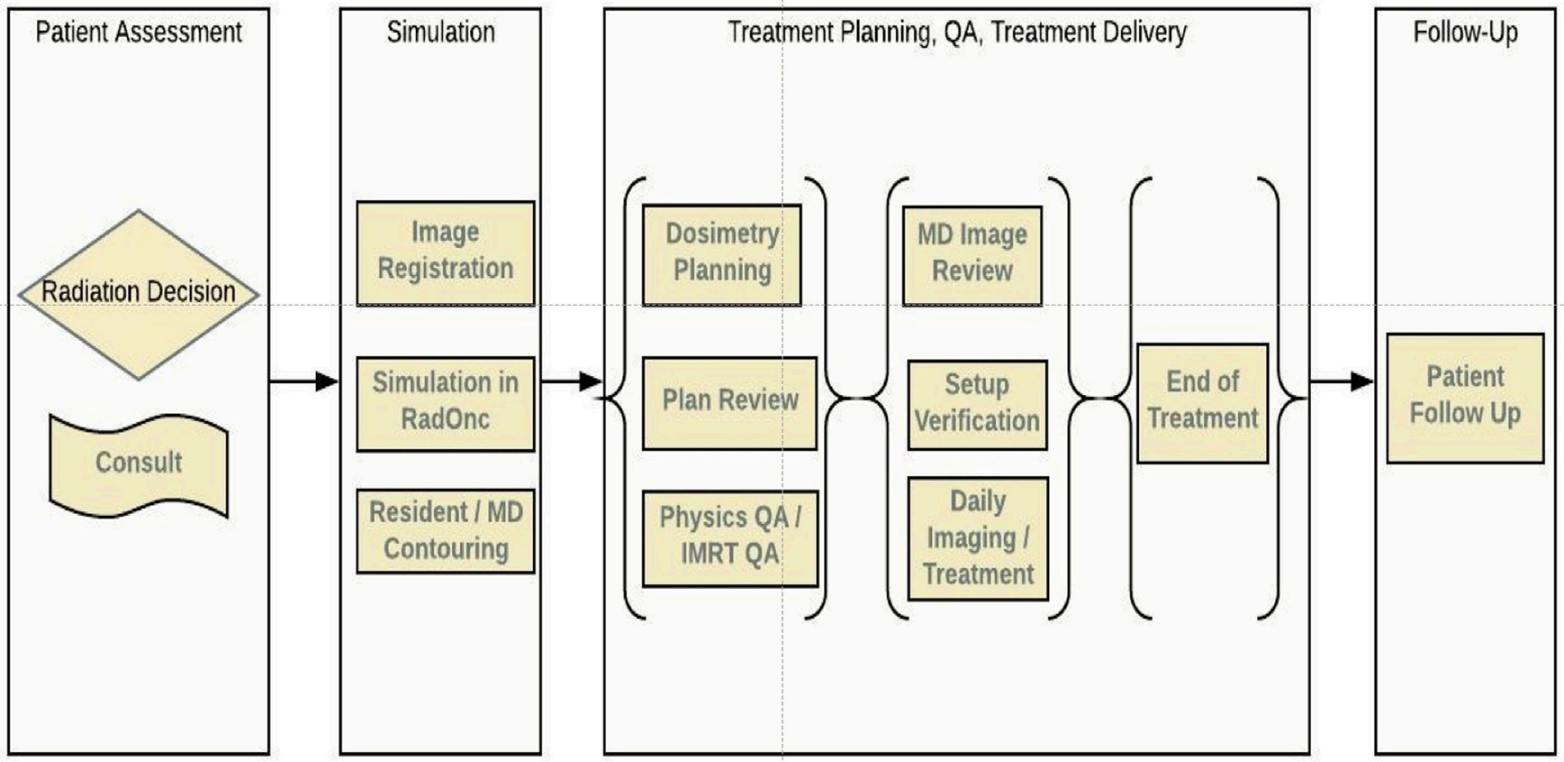
Radiotherapy workflow, from consult to follow-up.

In this article, we review the radiotherapy process from a workflow perspective, identifying specific areas where ML could improve the quality and efficiency of the current caregiving process for patients treated with radiation therapy. We have divided radiotherapy into six serial stages that encompass the entirety of treatment: patient assessment, simulation, planning, QA, treatment delivery, and follow-up, Figure 1. In each of these areas, we have identified open questions, emerging techniques, and possible directions concerning all stakeholders: patients, oncologists, physicists, dosimetrists, therapists, and nurses. Each stage is accompanied by a systematic assessment of opportunities, expectations, applicability, and limitations of various ML algorithms. While the impact a data-centric approach can have on improving the quality of treatment for cancer patients is clear, utilizing such a method will require a cultural shift at both the professional and institutional levels. We believe that this article will serve as a guide for both clinicians and researchers on those problems that must be addressed in a timely manner.

## Patient Assessment

The radiation oncology process begins at the first consultation. During this time, the radiation oncologist and patient meet to discuss the clinical situation, including the risks and benefits of treatment and the patient’s goals of care, to determine a treatment strategy. Useful information to assess the potential benefit of treatment includes tumor stage, mutational status or gene signatures (e.g., MGMT, Oncotype score), viral status (e.g., HPV), prior and current therapies, margin status if post-resection, ability to tolerate multimodality therapy, and overall performance status. Balanced with this are parameters that impact potential risk and tolerability of treatment including age, comorbidities, functional status, functioning of important organs, proximity between tumor and critical normal tissues, supportive care network, and ability to cooperate with motion management. All of these are features can be used to build predictive models of treatment outcome and toxicity. These models, then, can be used to inform physicians and patients to manage expectations and guide trade-offs between risks and benefit.

Having an ontology to identify and categorize the information available at this stage is important for any successful application of any predictive model (7). By contrast, current predictive models utilizing tumor control or normal tissue complication probability are neither subdivided nor categorized according to current state of a patient within the treatment timeline (8–12). Rather, they make use of a predetermined set of features, collected by individual investigators, that may have previously shown correlation to a particular clinical outcome (8–12). As a result, physicians are limited by scare and siloed data, making it often necessary to make informed guesses rather than data-driven decisions.

Many opportunities can be found for predictive models at the stage of initial consult. Here are a few practical examples in which a data-centric approach could improve decision making at the time of consultation:

(1) You are asked to see an inpatient who has a painful cervical spine metastasis. She will be discharged to hospice. What information may be helpful to determine whether to recommend RT? On one hand, radiotherapy can palliate her pain. However, she may not live long enough to benefit from treatment, but will have the discomfort associated with transfers and positioning for simulation/treatment, acute esophagitis, and pain flare. Narcotic management may be best for her, but how would we know? Models which predict time to pain relief, risk of toxicity, and overall survival would help optimize decision making at end-of-life, maximizing quality of life for the patient, and delivering high-value care (13).
(2) A patient with intermediate-risk prostate cancer is referred to discuss therapy. Treatment options for could include fractionated external beam radiotherapy, stereotactic body radiotherapy, brachytherapy, surgery, and other non-radiation approaches, without or without androgen-deprivation therapies. Shared decision making is crucial in this situation, since each type of therapy has inherent trade-offs with different side-effect profiles, which drives choice of therapy. A clinical support tool showing the balance between efficacy and side effects based on pre-treatment function and choice of therapy would be helpful for physicians and patients in shared decision making.
(3) A patient with hepatitis C cirrhosis and a single hepatocellular carcinoma is referred to consider treatment options. To determine whether to recommend SBRT over other treatments such as radiofrequency ablation or transarterial chemoembolization, information on liver function, suitability for anesthesia, and proximity to bowel, heart, gall bladder, central biliary tree is needed. Comprehensive ways to integrate tumor control and toxicity predictions from all treatment modalities would help the physician and patient to manage expectations and decide on a course of therapy. Within radiation oncology, there has been some work done to model individual radiation sensitivity to individualize and adapt therapy, though there is still much opportunity for richer predictive modeling using ML (14).
(4) A patient with early stage left-sided breast cancer had a lumpectomy with negative margins and comes to discuss adjuvant radiotherapy. How would you decide whether she would benefit from deep inspiration breath-hold or intensity-modulated radiotherapy rather than 3D conformal RT? Would proton therapy be beneficial for this patient? Since the complexity and cost is higher with more advanced technology, models to predict who would benefit would be helpful for technology selection and resource allocation (11, 15–18).

The delivery of models that could help with these scenarios will require a cultural shift in our profession toward standardization and collaboration. In this regard, new collaborative projects have begun in recent years, though participation is not yet widespread (12, 19, 20). In addition, while recently published task reports have aimed to standardize nomenclature in radiotherapy (21), it is equally important to develop standards for data collection. Due in part to the small datasets typically encountered in radiotherapy, the choice of algorithm in a specific application can produce differences of up to 32% in predicted outcome (22). It is also important to understand the goal of any modeling effort. If the goal is to assist physicians and patients reach the best decision, then a balance between interpretability of the results and accurate predictions is needed (23, 24). In this case, logistic regressions or decision trees are equally effective (23, 24). If accuracy is favored over interpretability, then tree base methods such as random forests or gradient boosting, and Support Vector Machines with kernel methods, consistently win most modeling competitions when structured data are analyzed (such as the type of data described above) (25, 26).

## Simulation

Once a physician and patient have decided to proceed with radiation therapy, the physician will place robust instructions for a Simulation, which is then scheduled. The order for simulation includes details about immobilization, scan range, treatment site, and other specifics necessary to complete the procedure appropriately. Patient preparation for simulation could include fiducial placement, fasting or bladder/rectal filling instructions, or kidney function testing for IV contrast. Special instructions are given for patients with a cardiac device or who are pregnant, and lift help or a translator is requested if necessary.

In most cases, a Simulation is scheduled after appropriate CT orders have been placed in the electronic medical record. Following completion and review of the CT simulation, the scan is exported to a planning system for the physician to contour tumor volumes and organs at risk (OARs). Sometimes a MR, PET and/or pre-operative scan is registered. With the OARs and tumor volumes contoured, the dosimetrists then begin designing a treatment plan based on specific physician instructions.

A good CT simulation is critical to the success of all subsequent processes, to achieve an accurate, high quality, robust, and deliverable plan for a patient. It is not uncommon that deficiencies at the time of CT simulation result in a need for a patient to return for a repeat CT, including insufficient scan range, incorrect IV contrast protocols, suboptimal immobilization, incorrect bladder/rectal filling, artifacts from internal hardware or those caused by the 4DCT process, lack of breath-hold reproducibility, and so on. Thus, focusing on the simulation, in particular, there are many questions that could be answered through ML algorithms to aid in decision making and overall workflow efficiency:

1. Will this patient benefit from IV contrast?
2. Will this patient be compliant with immobilization and motion management technique (e.g., compression or breath hold)?
3. Considering breathing patterns and other issues, will a 4DCT be beneficial for this patient?
4. Will this patient be able to tolerate the duration of the intended treatment (AP/PA vs. SBRT) and IGRT method (CBCT vs. kV-kV Orthogonal)?
5. Will this patient’s anatomy allow for standard immobilization for simulation and treatment?

Simulation is an area where the community has focused little effort on ML, with early work confined to predicting tumor motion (27–30). Additional emphasis, from both academic institutions and industry, can be expected in the future.

## Treatment Planning

The planning process starts by delineating both the target(s) and the OARs. While a number of commercial auto-segmentation algorithms exist, the underlying technology relies on an atlas-based strategy rather than utilizing ML. The performance of atlas-based segmentation tools depend highly on the type of structure, showing better results for high-contrast organs (e.g., lung) while struggling with soft tissue organs (e.g., pancreas) (31). By contrast, recent advances in computer vision, specifically around deep learning (6, 32), are particularly well suited for auto-segmentation (33–35). In deep learning, the algorithm is tasked to design the best features (higher order features) from the raw data as well to produce the classifiers (6). This is particularly important when human experts are unable to design proper features or quantify a given process, as in computer vision problems. An important limitation of the application of deep learning to segmentation is the limited size of the datasets available in radiation oncology. Because the algorithm is tasked to find the features as well as the classifier, deep learning models contain millions of parameters, and thus require more data than traditional ML algorithms. In applications in which deep learning has been successfully applied, the models have been trained with tens of thousands observations (4, 36). Although there are techniques to prevent overfitting when the number of parameters is larger than the number of observation points (transfer learning, dropout, early stopping), it remains to be demonstrated whether these algorithms can generalize to datasets on the order of a few hundred in size, even when the techniques mentioned above are used. In our opinion, the effective application of deep learning to segmentation requires training and validation on datasets across multiple institutions and multiple scanners.

Once the target volumes and OARs have been delineated, the planning process continues by (1) setting dosimetric goals for targets and normal tissues; (2) selecting an appropriate treatment technique (e.g., 3D, fixed beam IMRT, VMAT, protons); (3) iteratively modifying the beams/weights/etc., until the planning goals have been achieved; (4) evaluating and approving the plan. It is in this last step where most ML applications have been focused (37–43). While there techniques are typically referred to as knowledge-based planning (KBP), it is important to highlight that both current academic research and available commercial products are limited to predicting dose–volume histograms (DVHs) within accepted ranges. Several authors have shown the value that DVH prediction has in improving population based treatment plan quality and in the detection of outliers (44–47).

Similar gains in steps 1–3 highlighted above would be equally important. For example, while KBP can predict DVHs, the intrinsic trade-offs between dosimetric indices that must be considered in step 1 are not currently predicted. A more recent commercial product, Quick Match (Siris Medical, Redwood City, CA, USA), uses gradient boosting (the most accurate algorithm on expectation when structured data are available) to explore predictions in dosimetric trade-offs (17). This application, which is similar to a treatment planning Pareto solution but obtained before the treatment planning process, can facilitate communication between dosimetrist and physicians, establish individualized and achievable goals, and help physicians and patients decide the course of plan before embarking on the treatment planning process. In addition, by allowing the exploration of intrinsic trade-offs, it can also help to choose an optimal technique (e.g., photon vs. protons).

Once the dosimetric goals have been established and the technique chosen, automatic plan generation is also possible. Attempts have been made to solve various aspects of this problem, for instance, predicting the best beam orientations (48, 49). The larger task of automated treatment planning, however, is well suited for reinforcement learning. In this technique, widely used in games, self-driving cars, and other popular-culture applications, an algorithm learns to navigate a set of rules, given some constraints, by self-correcting its decisions. For example, one could use fundamental laws of radiation interaction to achieve certain dosimetric constraints. Essentially, the algorithm will take a decision (for instance, increase the weight of a given constraint) and learn from the simulator (the treatment planning system) whether the decision resulted in the right direction. Common to successful applications of reinforcement learning is the ability to generate synthetic data using a simulator (e.g., games). This technique, successfully used by Google Brain to develop an algorithm capable of beating a Go world champion (5), could provide performance at the level of our best dosimetrists if properly implemented. One challenge of achieving automatic planning using reinforcement learning lies in the close integration that this research endeavor will need with robust treatment planning systems. Therefore, it seems likely that an industry/academic partnership is best suited to achieve this goal. Summarizing then, in the future, we envision the planning process to happen fully automatically, from contouring to plan creation, with the human experts (dosimetrists, physicists, and physicians) evaluating, supervising, and providing QA to the given results.

## QA and Treatment Delivery

A number of aspects of a radiotherapy QA program, specifically in error detection and prevention, treatment machine QA, and time series analyses, are well suited to the application of ML (50–53). Li and Chan developed an application to predict the performance of linear accelerators over time (51). Valdes et al. developed ML applications to predict IMRT QA passing rates (52, 53) and to automatically detect problems with the Linac imaging system (50). Carlson et al. developed a ML approach to predict multi-leaf collimator positional errors (54). El Naqa developed system to detect anomalies in QA data (55). Finally, Ford et al. have developed a tool to quantify the value of quality control checks in radiation oncology (56). The ability of these algorithms to automatically detect outliers allows physicists to focus attention on those aspects of a process most likely to impact our patient care, as recommended in Task Group 100 (57).

Other important applications of ML include predicting planning deviations from the initial intentions and predicting the need for re-planning. Guidi et al. developed a ML-based tool to predict when head and neck patients treated with photons need re-planning (58). In a similar fashion, Tseng et al. used three deep neural networks to predict the need for treatment adaptation for lung patients (59, 60). Varfalvy et al. used relative gamma analysis and hidden Markov models to categorize patients based on deviations from the initial treatment plan to identify patients in need of re-planning. The need to predict proton patients who would benefit from a re-plan is even more relevant, though no publications exist in this setting to date. Deciding on the best algorithm for QA applications is critical for accurately predicting outcomes. While it is clear that each of the applications described above are important and useful, all remain within the domain of research and have not been made available commercially.

## Follow-Up

Machine learning also has the potential to change the way radiation oncologists follow patients treated with definitive therapy. Following surgery, the tumor may disappear on imaging, and tumor markers may quickly normalize. By contrast, the evolution of imaging changes (loss of enhancement, PET avidity, or diffusion restriction; stability or decrease in size) and response of tumor markers is gradual following radiotherapy. These features are monitored regularly over time, with qualitative changes complemented with clinical experience providing indication of therapeutic efficacy. Clearly, better models based on early assessment are needed to predict outcome, in time for treatment intensification with additional RT, early addition of systemic therapy, or application of a different treatment modality. In this regard, early work in the area of radiomics seems promising. In radiomics, quantitative features, including those based on size and shape, image intensity, texture, relationships between voxels, and fractal characteristics, are extracted to characterize an image. ML algorithms can then be deployed to correlate the image-based features with biological observations or clinical outcomes (59–64). The limited reproducibility of imaging systems both within and across institutions remains a significant challenge for radiomics (65, 66). And while the application of deep learning to image quantification has produced stellar results in other areas (67), it is important to understand that these techniques required thousands of data points even when transfer learning was used, which can prove challenging in radiation oncology, where datasets are limited.

## Conclusion

Machine learning is poised to impact the profession of radiation oncology, from patient consult to follow-up. While the excitement around ML and big data is well justified, many challenges remain, a number of which we have tried to describe above. There are also several broad challenges we will have to address as a field. The first is the creation and curation of large datasets. Although it is highly unlikely that robust models can be built with data from a single institution alone, the need to develop data sharing agreements can be a significant barrier to the development of these models. One potential solution to the challenges associated with multi-institutional data sharing is the use of distributed learning; the group at Maastricht University led by Philippe Lambin has been pioneering this approach (68, 69). Standardization of the data collection process is also essential for training models using datasets from multiple institutions. In addition, it is important to highlight that distributed learning and transfer learning are part of the larger discipline of ML and to maximize learning from all centers while customizing the solution to each, mathematical guarantees and constraints are necessary to ensure algorithms do not “forget” previous seen datasets (70). Tailoring these algorithms to radiation oncology needs will also be an active research area in the future. Quality of data is also of paramount importance as no ML algorithm today can fix problems contained within the training data. In this regard, interpretability of algorithms used (e.g., ability for humans experts to understand reasons behind a prediction) will play an important role to avoid preventable errors (23).

Finally, training of our workforce and updating our educational curriculums will be increasingly important. As with any algorithm that we use in radiation oncology today (e.g., dose calculation, deformable registration), ML algorithms will need commissioning and QA. Clinicians will need to learn to interpret and understand the limitations of any results. The field of radiation oncology is highly algorithmic and data-centric, and while the road ahead is filled with potholes, the destination holds tremendous promise.

## Author Contributions

All authors were involved with the conception and design, manuscript writing, and final approval of the manuscript.

## Conflict of Interest Statement

The authors declare that the research was conducted in the absence of any commercial or financial relationships that could be construed as a potential conflict of interest. The reviewer MC declared a past collaboration with several of the authors GV, TS to the handling Editor.
